# Correcting for the effects of natural abundance in stable isotope resolved metabolomics experiments involving ultra-high resolution mass spectrometry

**DOI:** 10.1186/1471-2105-11-139

**Published:** 2010-03-17

**Authors:** Hunter NB Moseley

**Affiliations:** 1 Department of Chemistry, Center for Regulatory and Environmental Analytical Metabolomics, University of Louisville, Louisville, Kentucky USA

## Abstract

**Background:**

Stable isotope tracing with ultra-high resolution Fourier transform-ion cyclotron resonance-mass spectrometry (FT-ICR-MS) can provide simultaneous determination of hundreds to thousands of metabolite isotopologue species without the need for chromatographic separation. Therefore, this experimental metabolomics methodology may allow the tracing of metabolic pathways starting from stable-isotope-enriched precursors, which can improve our mechanistic understanding of cellular metabolism. However, contributions to the observed intensities arising from the stable isotope's natural abundance must be subtracted (deisotoped) from the raw isotopologue peaks before interpretation. Previously posed deisotoping problems are sidestepped due to the isotopic resolution and identification of individual isotopologue peaks. This peak resolution and identification come from the very high mass resolution and accuracy of FT-ICR-MS and present an analytically solvable deisotoping problem, even in the context of stable-isotope enrichment.

**Results:**

We present both a computationally feasible analytical solution and an algorithm to this newly posed deisotoping problem, which both work with any amount of ^13^C or ^15^N stable-isotope enrichment. We demonstrate this algorithm and correct for the effects of ^13^C natural abundance on a set of raw isotopologue intensities for a specific phosphatidylcholine lipid metabolite derived from a ^13^C-tracing experiment.

**Conclusions:**

Correction for the effects of ^13^C natural abundance on a set of raw isotopologue intensities is computationally feasible when the raw isotopologues are isotopically resolved and identified. Such correction makes qualitative interpretation of stable isotope tracing easier and is required before attempting a more rigorous quantitative interpretation of the isotopologue data. The presented implementation is very robust with increasing metabolite size. Error analysis of the algorithm will be straightforward due to low relative error from the implementation itself. Furthermore, the algorithm may serve as an independent quality control measure for a set of observed isotopologue intensities.

## Background

Application of mass spectrometry to stable isotope tracing experiments for the elucidation of glucose dates back to at least the early 1980's [1,2]. The general scheme for these experiments is to supply a labeled precursor such as uniformly-labeled ^13^C glucose ([U-^13^C]-glucose) to a bacterial culture, tissue culture, or a whole multicellular organism and then extract a set of cellular or excreted metabolites for analysis [3,4]. For identified metabolites, specific patterns of isotopologues are usually observed, which are then interpreted within the context of known cellular metabolic pathways [3-5]. Recently, we applied this technique to elucidate specific aspects of lipid metabolism [6].

The ultra-high resolution capability of Fourier transform-ion cyclotron resonance-mass spectrometry (FT-ICR-MS) makes it possibility to identify simultaneously hundreds, if not thousands, of metabolites from crude cell extracts without the need for chromatographic separation [6]. The better than 1 ppm mass accuracy of state-of-the-art FT-ICR-MS is often high enough to provide mass-to-charge ratios (m/z) down to the 3^rd ^and 4^th ^decimal place for metabolites less than a few thousand Daltons. This is accurate enough to distinguish relativistic mass differences between expected isotopes of CHONPS elements and unambiguously determine the isotope-specific molecular formula of an individual peak. Furthermore, the FT-ICR-MS's high mass resolution allows for the direct detection or deconvolution of individual isotopologues or mass-equivalent sets of isotopomers for a given metabolite.

Isotopologue identification and quantification of thousands of metabolites in these metabolomic experiments can provide a wealth of data for modeling the flux through metabolic networks. But before isotopologue intensity data can be properly interpreted, the contributions from isotopic natural abundance must be factored out (deisotoped). This is a computationally expensive and analytically intractable problem for data from lower mass resolution spectrometers where individual isotopically-resolved isotopologues cannot be distinguished [7]. In these instances, numerical methods have been employed to approximate and subtract the contributions from isotopic natural abundance [4,7-9]. Some of these calculations are aimed at a different deisotoping problem, namely identifying the related isotopologues and calculating the monoisotopic mass from its isotopic mass distribution [10,11]. Fortuitously, with the isotope-resolved isotopologue peaks from FT-ICR-MS histograms, we can pose a similar but distinct problem that allows for the derivation of a computationally tractable analytical solution. In addition, isotopologues derived from the same molecule (or very similar set of molecules) neatly handle peak intensity referencing issues by providing a natural internal reference.

## Results

### Derivation of the analytical solution

Equation 1 represents the relative distribution of carbon isotopologues from natural abundance only, as a sum of multinomial coefficients multiplied by the intensity of I_M+0_, the theoretically untainted ^12^C monoisotopic peak. The terms being summed are similar in form to those presented in Snider, 2007. I_M+i;NA _is the expected intensity of the i^th ^isotopologue peak representing i additional nucleons. NAx_C _is the fractional natural abundance of the ^X^C isotope. C_Max _is the number of carbons in the molecule. The multinomial coefficients, derived from the multinomial theorem with 3 variables represent the number of possible isotopomers of identical mass for a molecule with C_Max _carbons given 3 isotopes of carbon: ^12^C, ^13^C, and ^14^C.

Isotopologue peaks containing ^14^C are typically not observed, since the isotope is very rare. Moreover, due to the very high mass resolution in FT-ICR-MS histograms, isotopologue peaks representing molecules comprised exclusively of the major isotope of CHONPS elements (expected elements for biological systems) along with ^13^C, are completely resolved/deconvoluted and identified. Thus, we can ignore the contributions from ^14^C and from minor isotopes of all other elements excluding carbon. This simplifies the calculation to a *single term with a binomial coefficient *(binomial term) shown in Equation 2, where NA13_C _≈ 0.01109. The binomial coefficient represents the number of possible isotopomers of identical mass for a molecule with C_Max _carbons given only 2 isotopes of carbon: ^12^C and ^13^C.(2)

At natural abundance, each peak, I_M+i;NA_, is directly related to the theoretically untainted ^12^C monoisotopic peak, I_M+0_, that has a fractional intensity of 1 when dividing by the sum of isotopologue intensities. However, once ^13^C is incorporated into the molecule from a labeling source, the calculation of the contributions from natural abundance becomes more complex [8,9]. The effects of ^13^C natural abundance now depend on the amount of ^13^C label already present. With each ^12^C/^13^C isotopologue resolved in the FT-ICR-MS histogram, we can use a series of binomial terms to accurately describe and correct for ^13^C natural abundance. Equation 3 shows the basic form of these binomial terms as B_C_(n, k) where k represents the total number of ^13^C carbons present, n represents the number of ^13^C carbons due to incorporation from a labeling source, and k-n is the number of ^13^C carbons due to natural abundance. The binomial coefficient in Equation 3 enumerates the number of ways that k-n ^13^C carbons can be incorporated into the molecule when n carbons are already labeled with ^13^C. Equation 4 shows the first series needed in the correction, B_C_sum(n) which represents the fraction of I_M+i _intensity that is converted to other isotopologues due to the effects of natural abundance.(3)

Equation 5 shows the full correction as the original isotopologue intensity minus natural abundance contributions based on lower mass untainted isotopologue intensities. Division by the fractional intensity, 1 - B_C_sum(i), compensates for natural abundance effects that lower the intensity of the given isotopologue. As illustrated in Table 1, Equation 5 must be applied in a sequential fashion starting with i = 0, since the results of each step are needed in subsequent steps. In other words, the natural abundance corrected intensities of isotopologues with lower ^13^C incorporation from labeling are needed to calculate the natural abundance correction of isotopologues with higher ^13^C incorporation from labeling.(5)

**Table 1:** Sequential correction of ^13^C natural abundance effects in a four-carbon example

**I**_**M+i **_**= **^**b**^**Value = (**^**c**^**I**_**M+i;NA **_**- ΣI**_M+x _*B_**C**_**(x, i))/(1 - B**_**C**_**sum(i))**

I_M+0 _= 1.00 = (0.956)/(1.00 - 4.36E-2)
I_M+1 _= 1.00 = (1.01 - 1.00 * 4.29E-2)/(1.00 - 3.29E-2)
I_M+2 _= 0.00 = (3.33E-2 - 1.00 * 7.22E-4 - 1.00 * 3.25E-2)/(1.00 - 2.21E-2)
I_M+3 _= 0.00 = (3.70E-4 - 1.00 * 5.40E-6 - 1.00 * 3.65E-4 - 0.00 * 2.19E-2) /(1.00 - 1.11E-2)
I_M+4 _= 0.00 = (1.38E-6 - 1.00 * 1.51E-8 - 1.00 * 1.36E-6 - 0.00 * 1.23E-4 - 0.00 * 1.11E-2)/(1.00 - 0.00)

The example involves a four-carbon molecule with ^13^C-labeling involving a single carbon 50% of the time. Each row represents a single application (step) of Equation 5. All numbers rounded to three significant figures.

^b^Corrected isotopologue values. The use of these values in subsequent steps is highlighted in bold.

^c^Contaminated isotopologue values.

Since ^15^N incorporation can be distinguished from ^13^C incorporation due to the very high mass resolution in FT-ICR-MS histograms, it takes only a trivial conversion of Equations 3, 4, and 5 to handle labeling in ^14^N/^15^N isotopologues. We simply replace N_Max _for C_Max _and NA15_N _for NA13_C_. However, handling all of the mixed ^14^N/^15^N/^12^C/^13^C isotopologues that arise from simultaneous ^13^C and ^15^N labeling requires a series of two binomial terms multiplied together as shown in Equations 6 and 7. Given the peaks are isotopically resolved, there are CMax * NMax separate observable isotopologues, whose intensities are represented by I_M+i, j;NA_. The multiplied binomial terms, B_C_(x, i) * B_N_(y, j), describe the combined effects from both carbon and nitrogen natural abundance.(6)

A version of each equation in larger fonts is available in Additional file 1.

### Implementation of the algorithm

**Figure 1:**
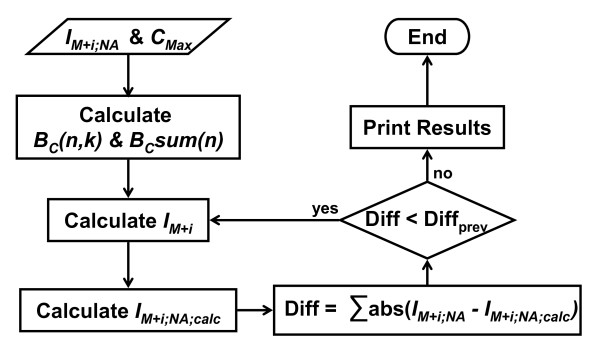
**Flowchart of **^**13**^**C natural abundance correction algorithm**. Starting with isotopologue intensities and C_Max _given as input, the algorithm precalculates needed binomial coefficients. Next, the algorithm calculates the corrected intensities and uses them to calculate the natural abundance contaminated intensities. Then the algorithm compares the observed and calculated contaminated intensities and only continues for another iteration if an improvement is made.

We implemented Equations 3, 4, and 5 as an iterative algorithm in the Perl programming language [Additional file 2]. Iteration allows the algorithm to partially compensate for missing (zero intensity) isotopologues. The algorithm (Figure 1) starts with C_Max _and the observed ^12^C/^13^C isotopologue intensities contaminated by contributions from ^13^C natural abundance. Based on C_Max_, the algorithm precalculates the binomial coefficients needed in later steps using Equations 3 and 4. During each iteration, the algorithm performs three steps. In step 1, the algorithm calculates the set of uncontaminated ^12^C/^13^C isotopologue intensities using Equation 5 and the observed intensities supplemented with calculated contaminated intensities for missing isotopologues. From Equation 5, it is apparent that this must be done in ascending mass order starting with I_M+0_. Sometimes, small negative uncontaminated intensities arise from errors in the observed intensities. These negative intensities are flattened to zero, since they have no basis in reality. Next, the algorithm renormalizes the uncontaminated intensities based on the sum of observed intensities. This is required since missing isotopologues were supplemented with calculated values and negative intensities are flattened to zero. In step 2, the algorithm calculates the set of contaminated intensities based on the uncontaminated set by solving for I_M+i;NA _in Equation 5. In step 3, the algorithm calculates the absolute difference between observed and calculated contaminated intensities. If this difference decreases, the algorithm performs another iteration until no more improvement is seen. Finally, the algorithm prints the results and ends.

### Testing the implementation

**Figure 2:**
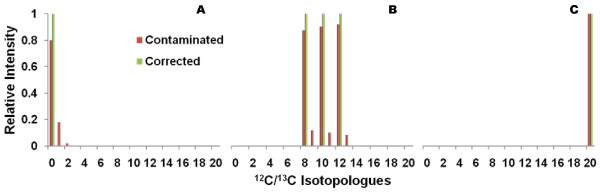
**Simulated **^**12**^**C/**^**13**^**C isotopologues of 20 carbon metabolite**. In all 3 charts, the red bars represent the relative isotopologue intensities with contributions (contamination) from ^13^C natural abundance. The green bars represent the corrected isotopologue intensities with this contamination removed. Chart A shows the expected isotopologues of a metabolite with no additional ^13^C-labeling present. Chart B shows the expected isotopologues of a metabolite with equal amounts of ^13^C-labeling for 8, 10, and 12 carbons. Chart C shows the expected isotopologues of a metabolite with 100% ^13^C-labeling.

We created several sets of simulated isotopologues (test sets) with varying levels of ^13^C-labeling and added the expected contributions (contamination) from ^13^C natural abundance by solving for I_M+i;NA _in Equation 5. We then tested the implementation with these test sets. Figure 2 shows the results for three of these test sets of a hypothetical metabolite with 20 carbon atoms. The ^13^C natural abundance contaminated intensities are in red and corrected intensities in green. The red bars in Figure 2A represents the expected observed isotopologue intensities when no ^13^C-labeling is present. This naturally collapses into a single green ^12^C monoisotopic peak with correction. Figure 2B shows the contaminated and corrected isotopologue intensities when equal amounts of ^13^C-labeling for 8, 10, and 12 carbons are present. There is a tapering phenomenon observed in the contaminated intensities due to the fact that the number of carbons affecting the intensities decreases with increasing amounts of ^13^C-labeling. Equation 3 captures this phenomenon within its binomial coefficient where it is further demonstrated in Figure 2C with natural abundance having no effect on a metabolite with 100% ^13^C-labeling.

The implementation is also quite efficient even in an interpreted programming language like Perl. 10,000 repetitions of this algorithm for all 3 simulated test sets took only 17 seconds on a single core of an Intel T7200 Core 2 Duo mobile processor with 2GB of RAM and running release 5.3 of the RedHat Enterprise Linux operating system. The implementation is also very accurate. Given the perfect data in these three simulated test sets, the largest error was 4.12 × 10^-16 ^seen in the I_M+1 _corrected intensity for the test set representing no ^13^C-labeling (Figure 2A). Furthermore, the implementation appears quite robust since the relative error actually decreases as the number of carbons (C_Max_) increases. At a C_Max _= 100, the relative error is 6.77 × 10^-17^. This implementation does have some numerical limitations, for example, the C_Max _must be less than 270 carbons due to all numerical quantities being represented as double precision (64 bit) floating point numbers. However, this limitation is easily overcome by using higher precision floating point numbers.

### Application to phosphatidylcholine 34:1 observed isotopologue intensities

**Figure 3:**
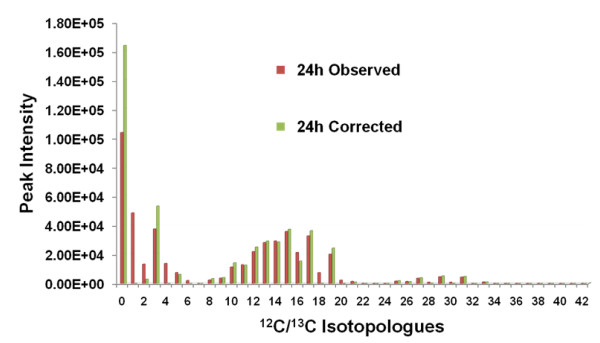
**^12 ^C/^13 ^C isotopologues of phosphatidylcholine 34:1**. Bar chart of ^13^C natural abundance contaminated (red) and corrected (green) sets of isotopologues. The lipid metabolite contains 34 carbons in the 2 fatty acid chains, 3 carbons in the glycerol, and 5 carbons in the choline head group.

Figure 3 shows the two sets of ^12^C/^13^C isotopologue intensities for phosphatidylcholine 34:1 (34 carbons in 2 fatty acid chains with only 1 double bond), with ^13^C natural abundance contaminated intensities in red and corrected intensities in green. The algorithm converged within 8 iterations to produce the corrected intensity results. In comparing the contaminated and corrected intensities, the most significant changes are seen in isotopologues 0-4 and 16-20. The drastic drop in I_M+1_, I_M+2_, and I_M+4 _isotopologues make the incorporation of ^13^C-labeled glycerol much clearer. Also, the drop in I_M+16_, I_M+18_, and I_M+20 _isotopologues supports the expected incorporation of ^13^C-labeled acetyl groups in the fatty acid chain biosynthesis.

## Discussion and Conclusions

Overall, correcting for the effects of natural abundance makes interpretation of isotopologue intensities from stable isotope tracing experiments easier within the context of cellular metabolism. Such a correction is required before using more quantitative methods of interpretation. Since the relative error is virtually zero with perfectly simulated data and the algorithm is very robust with increasing C_Max_, the accuracy of this correction is really only limited by the error in the isotopologue intensities themselves. Thus, the propagation of data error through this algorithm should be straightforward to analyze and quantify. Moreover, from Equation 5 it is evident that effects from natural abundance significantly link together groups of observed isotopologue intensities. This difference between calculated and observed intensities should be highly sensitive to the error in a set of isotopologue intensities. Therefore, this difference should be usable as an independent check on the quality of the observed set of isotopologue intensities. Such a quality control check would be especially useful when it is not possible or practical to repeat experiments or to determine whether additional experiments are necessary.

## Methods

### Cell Culture and FT-ICR-MS

We separated glycerophospholipids from crude cell extracts derived from MCF7-LCC2 cells in tissue culture after 24 hours of labeling with uniformly labeled ^13^C-glucose. We analyzed the sample on a hybrid linear ion trap 7T FT-ICR mass spectrometer (Finnigan LTQ FT, Thermo Electron, Bremen, Germany) equipped with a TriVersa NanoMate ion source (Advion BioSciences, Ithaca, NY) as described elsewhere [6].

## Authors' contributions

The author derived the analytical solution, implemented the algorithm, tested the implementation, applied the algorithm to the lipid metabolite experimental data, and wrote the manuscript.

## Funding

Supported in part by NSF EPSCoR grant #EPS-0447479.

## Supplementary Material

Additional file 1 **Equations**. This file contains all equations in Word 2007 format. Click here for file

Additional file 2 **Perl program implementing the algorithm**. Perl program implementing the algorithm displayed in a ASCII text file. Click here for file
