# Supplementary material for: Correcting for the effects of natural abundance in stable isotope resolved metabolomics experiments involving ultra-high resolution mass spectrometry
Source: BMC Bioinformatics. 2010 Mar 17;11:139. doi: 10.1186/1471-2105-11-139 (PMC2848236; doi:10.1186/1471-2105-11-139)
Supplement: Additional file 1 — Equations. This file contains all equations in Word 2007 format. [file 1471-2105-11-139-S1.DOCX]

Eq. 1

$$I_{M+i;NA}=I_{M+0}\left[ \sum_{j,k}^{\begin{aligned} j,k\geq0 \\ j+2k=i \end{aligned}} \left( \begin{matrix} C_{Max} \\ j,k,C_{Max}-j-k \end{matrix} \right){{NA}_{{}^{12}C}}^{C_{Max}-j-k}{NA}_{{}^{13}C}^{j}{NA}_{{}^{14}C}^{k} \right]$$

Eq. 2

$$I_{M+i;NA}=I_{M+0}\left( \begin{matrix} C_{Max} \\ i \end{matrix} \right){{(1-NA}_{{}^{13}C})}^{C_{Max}-i}{NA}_{{}^{13}C}^{i}$$

Eq. 3

$$B_{C}\left( n,k \right)=\left( \begin{matrix} C_{Max}-n \\ k-n \end{matrix} \right){(1-{NA}_{{}^{13}C})}^{C_{Max}-k}{NA}_{{}^{13}C}^{k-n}$$

Eq. 4

$$B_{C}sum\left( n \right)=\sum_{k=n+1}^{C_{Max}} B_{C}(n,k)$$

Eq. 5

$$I_{M+i}=\frac{I_{M+i;NA}-\sum_{x=0}^{x<i} I_{M+x}*B_{C}(x,i)}{1-B_{C}sum(i)}$$

Eq. 6

$$I_{M+i,j}=\frac{I_{M+i,j;NA}-\sum_{y=0}^{y<j} \sum_{x=0}^{x<i} I_{M+x,y}*B_{C}\left( x,i \right)*B_{N}(y,j)}{1-B_{C,N}sum(i,j)}$$

Eq. 7

$$B_{C,N}sum\left( n,m \right)=\sum_{k=n+1}^{C_{Max}} \sum_{l=m+1}^{N_{Max}} B_{C}(n,k)B_{N}(m,l)$$
